# Suspected MRI associated burn injuries in dogs

**DOI:** 10.3389/fvets.2025.1688254

**Published:** 2026-01-05

**Authors:** Jamie L. Peyton, William T. N. Culp, Rich Larson, S. Jason Peters, Christine M. Toedebusch, Eric G. Johnson

**Affiliations:** 1 School of Veterinary Medicine, One Health Institute, University of California, Davis, Davis, CA, United States; 2 College of Veterinary Medicine, Washington State University, Pullman, WA, United States; 3 Department of Surgery and Radiology, School of Veterinary Medicine, University of California, Davis, Davis, CA, United States; 4 School of Veterinary Medicine, Veterinary Medical Teaching Hospital, University of California, Davis, Davis, CA, United States; 5 Department of Molecular Biosciences, School of Veterinary Medicine, University of California, Davis, Davis, CA, United States

**Keywords:** burn, wound, MRI, canine, MRI safety, thermal injuries

## Abstract

Magnetic Resonance Imaging (MRI) is widely utilized in veterinary medicine for its diagnostic accuracy and safety profile. However, thermal injuries secondary to MRI are a significant adverse event that may be under recognized by veterinary clinicians. This study investigates five canine cases of presumed MRI-induced burns with a distinctive linear pattern, ranging from superficial to full-thickness, at a single veterinary institution. The underlying mechanisms for these thermal injuries was most likely resonant circuit heating or the antenna effect, exacerbated by patient positioning and improper insulation of ECG leads. Clinical implications included delayed wound recognition, extended healing times, and substantial complications such as impaired mobility and delayed neurological rehabilitation. The findings underscore the necessity for improved MRI safety protocols, particularly regarding patient monitoring and positioning during MRI procedures in veterinary practice.

## Introduction

Magnetic resonance imaging (MRI) is a valuable tool in veterinary diagnostic imaging and is generally regarded as a safe diagnostic tool especially given that ionizing radiation is not employed by this modality. However significant thermal injury can occur as an adverse event in patients undergoing MRI studies. Burns ranging from superficial to full thickness account for over two-thirds of the reported MRI-related injuries. These thermal injuries induced by MRI have been directly associated with electrocardiogram (ECG) monitoring equipment, pulse oximetry equipment, contact with imaging coils, skin to skin contact, and more bizarre associations such as ferromagnetically active tattoo ink and transdermal patches (1–3). These associations are described in the US Food and Drug Administration (FDA) “Center for Devices and Radiological Health” files and have been documented in a myriad of case reports in people. Universal preventative protocols in human medicine have been recommended to help prevent these injuries (4–9). It should be noted that despite these recommendations the number of MRI associated RF burns has increased over the past few decades with the FDA reporting 419 cases in 1997–2009 and 849 cases in 2008–2017. It is theorized that this may be due to increase field strength magnets with increased availability of 3 Tesla MRI units (7, 8).

The three most widely accepted mechanisms of MRI burns are electromagnetic induction heating, resonant circuit heating, and the antenna effect.

Electromagnetic induction heating is based on Michael Faraday’s law of induction which states that “The electro-motive force (emf) induced in a circuit is directly proportional to the time rate of change of magnetic flux through the circuit.” These principals are commonly used today in transformers to step up or step-down voltage and the biproduct of this change is heat. In the MRI bore, inductive heating with varied gradient magnetic fields and electromagnetic radiofrequency (RF) fields in conjunction with conductors can cause electrical currents to flow and generate heat. Burns can occur at areas of high resistance (e.g., a metal to skin interface) where they cause heat (2, 10).

Resonant circuit heating is a specific type of induction heating through which maximum current is induced as a circuit arrives at its resonant frequency where it can absorb and release energy. The specific frequency at which the resonance occurs can be tuned by altering the inductance or capacitance of the material (10). Experimentally this type of MRI heating can produce a temperature increase as much as 61.1 ± 0.01 degrees C and is directly linked to RF induced currents (2). Wire leads in the MRI bore can act as a resonant circuit. A looped or coiled metallic lead in contact with the patient’s skin surface may cause this type of burn. Radiofrequencies can theoretically generate currents and thus heat via electromagnetic induction in any conductive material in a “closed loop” (11). This can occur with cables looped upon themselves, cables crossing over and touching other cables, and cables in contact with the RF coils in more than one location (9). Importantly, skin is conductive, and these “closed loops” can form with skin-to-skin contact or skin contact with the coils and subsequently lead to burns (2, 10, 11).

The antenna effect is a result primarily of the electrical component of RF pulses interacting with a length of wire or cable. When this occurs the RF radiation enters the cable or wire and a subsequent current is generated (3). This phenomenon generates heat especially at resonance which is achieved when the antennae length is approximately one half of an RF wavelength long and experimentally can achieve a maximum temperature of 63.5 ± .01 °C. The heat generated is concentrated at the tip of a wire or metallic rod (2). In air the calculated length of antenna heating at 1.5 T is 220 cm which is one half the wavelength at 64 MHz (2). This value drops as we increase the field strength to 3 T with the calculated length for maximum antenna heating being 117 cm given the increase in frequency to 128 MHz. When dealing with soft tissues and saline phantoms this relationship changes since the wavelength decreases and in saline a wire implant length of as short as 25 cm can represent the resonance frequency at 1.5 T (12). The complexity of MR and RF interactions and heating of implants, and cables does not end here. The configuration (coiled vs. straight) and location in the body (superficial vs. deep) can all effect the relative heat generated when interacting with RF at a specific field strength (13).

MRI burns occurring with monitoring equipment including ECG leads and pulse oximetry can theoretically be traced to both electromagnetic induction heating and the antenna effect (10). This is mainly due to the variable nature of how the leads are placed on the patients. After MRI burns are reported, it is often difficult to trace back the exact position of the leads on the patients. In people, FDA reports have indicated that MRI coils are causal in burn injuries; however, drawing a conclusion on what physical mechanism in involved in the formation of the burn is difficult as coil specific information is often not provided (10). In people it also is common that skin to skin contact with the MRI can lead to RF associated thermal burns and direct skin to skin contact should be avoided (14).

In veterinary medicine, there is a dearth of published reports of clinically associated MRI burns despite the known risk with this imaging modality. Only a single abstract documenting first-degree burns associated with ECG electrodes, and a single case report of suspected skin to skin contact RF burns have been reported in dogs and cats (15, 16). These cases showed injuries that required minimal treatment. To gain more insight into the prevalence and severity of MRI-associated burns in small animal veterinary medicine, this study retrospectively examined medical records from a single institution over a 20-year period. We identified five cases of suspected MRI burns with burn grades ranging from superficial (first degree) to full thickness with bone exposure (third+degree burns). While MRI-associated burns were uncommon in this study, our data demonstrate that they can cause significant morbidity and highlight the need for great care when positioning patients for scanning.

## Materials and methods

The electronic medical records database at the William R. Prichard Veterinary Medical Teaching Hospital was retrospectively searched from 2000 to 2024 for “MRI,” “burns,” and “wounds.” This search yielded 432 patient records. These cases were evaluated for inclusion based on wound appearance, timeline, and features compatible with MRI burns. Some cases specifically were identified as suspected MRI burns. For inclusion, cases must have: (1) had an MRI during their current patient visit (within 14 days of wound discovery), (2) no other identifiable cause for the wound, and (3) wound features compatible with published data on MRI burns including the presence of hyperemia, swelling, and development of eschar in areas not associated with external heating devices. Patient records that met the inclusion criteria were then evaluated for the following: signalment, presenting complaint, date of MRI, surgery (if any) performed, date of wound appearance, wound characteristics and progression, days in hospital, treatments administered, and outcome.

## Results

Five cases met inclusion criteria. All cases were dogs with ages ranging from 4 to 8 years old (median 7 years) and weighed 7.8–49.7 kg (median 32.7 kg) (Table 1). All dogs body condition score was between 5 and 6 (Purina 9-Point Body Condition System) No cats met inclusion criteria. Four of the dogs were considered large breeds (Doberman Pinscher *n* = 2, Bernese Mountain Dog *n* = 1, and Great Dane *n* = 1) and one was a small breed (Dachshund), with 3 spayed females and 2 castrated males. In this study period there were 14,000 MRI exams performed, resulting in a 0.035% prevalence of MRI burns occurring in dogs and cats.

**Table 1:** Characteristics of canine patients with MRI burns.

Breed	BW (Kg)	MRI time (min)	Emergency after hours MRI	Swelling noted (days post MRI)	Open wound noted (days post MRI)	Location	Size (cm)	Depth	Epithelization (days)
Great Dane	47	40	No	12	12	Bilateral cubital	L-8×4 cm R-6 × 5 cm	SPT	14
Doberman	32.7	19	Yes	14	14	Right cubital	8 × 3 cm	FT (bone)	58 (with delayed primary closure)
Bernese Mountain Dog	49.7	45	Yes	Left-1 Right-6	Left-6 Right-12	Bilateral cubital	L-30 × 6 cm R-12 × 4 cm	FT (bone)	56 (with delayed primary closure)
Doberman	27	33	Yes	4	6	Left cubital	13 × 4 cm	FT (bone)	45 (with delayed primary closure)
Dachshund	7.8	23	Yes	1	1	Left thigh	8 × 3 cm	FT (SQ)	N/A

SPT, superficial partial thickness; FT, full thickness.

The presenting complaint (reason for undergoing MRI scanning) was tetraparesis in the four large breed dogs and paraplegia in one small breed dog. All dogs were placed under general anesthesia using standard institutional protocols and overseen by a board-certified veterinary anesthesiologist. Standard anesthetic monitoring was completed using MRI conditional equipment including ECG, non-invasive blood pressure, pulse oximetry and capnography. All patients were imaged on a 1.5 T system (GE Signa 1.5 T Horizon HiSpeed LX 9.1 MR System GE Healthcare, Chicago, IL). Four of the five dogs were imaged using a split head coil (GE 1.5 T Split Head Coil Assembly, 8 channel, receive only, GE Healthcare, Chicago, IL) positioned over the cervical vertebral column in conjunction with pathologic neuroanatomic localization and the remaining dog (Dachshund) was imaged using the GE MRI 1.5 T 8-Channel, receive only, CTL Coil (GE Healthcare, Chicago, IL) over the thoracolumbar vertebral column in conjunction with pathologic neuroanatomic localization (GE Healthcare, Chicago, IL). All dogs were imaged in dorsal recumbency. Transverse and parasagittal T1 and T2 weighted sequences of the spinal cord regions of interest were obtained in all patients. The average time for the MRI was 32 min (range 19–45 min). Four out of the five dogs had their MRI during emergency afterhours while only one was during regular operating hours.

All the dogs imaged with the split head coil had wounds along the cubital region (2 bilateral, 2 unilateral). The single dog imaged with the spine coil had a wound over the caudal thigh musculature. All the wounds had a marked distinctive linear pattern and were noted on average at 4 days post MRI (range 1–12 days). Localized swelling and erythema were observed in all dogs. In two dogs soft tissue swelling was initially noted which then progressed to full thickness (bone) wounds. Eschars were noted in the 4 dogs with full thickness burns on average at 10.5 days (range 4–19 days) with three dogs having wounds which progressed down to visible bone. The average wound size was 12 cm in length (range 6–30 inches) by 4 cm in width (range 3-6 cm). The data collected on these wounds is further summarized in Table 1.

All dogs were treated with daily wound cleansing using sterile saline and 0.024% hypochlorous acid. A combination of topical primary wound dressings, including calcium alginate, medical grade honey, granulated sugar, and hydrogel were also applied and followed with standard of care secondary dressings (non-adherent bandage, cast padding, roll gauze, vetwrap). One out of the four dogs with full thickness burns also was treated with a closed vacuum system. Three dogs with full thickness burns, down to the level of bone, were taken to surgery for delayed primary wound closure after a healthy granulation bed was observed (tertiary intention), while the other dog with full thickness down to the subcutaneous tissue healed via second intention. Wound healing, defined as time to full epithelialization, occurred within 14 days for the dog with the superficial partial thickness burn. Three out of four dogs with full thickness burns took an average of 53 days (range 45–58 days) to heal, with the fourth dog lost to follow up. All dogs survived to discharge (range 11–80 days; median 13 days).

Long-term follow-up was available for three dogs with full thickness burns that were continued as outpatients until complete wound healing; the fourth dog continued care with the referring veterinarian. All dogs with full thickness cubital wounds (n = 3) had substantial scarring and contracture of the soft tissues, which resulted in loss of elbow joint mobility and chronic thoracic limb lameness.

## Discussion

According to the FDA, the number of MRI burn cases are increasing and not decreasing; highlighting the importance of recognizing this potential complication. There were 419 cases reported in 1997–2009 and 849 cases reported in 2008–2017. This increase in cases may be related to the greater availability and accessibility of 3 T units (7, 8). Yet the most common causes include contact with a conductive object, contact with the scanner bore, and skin-skin contact. The severity of the burns range from mild superficial burns to severe full thickness burns leading to amputations; the most common burns were partial thickness involving the dermis (1, 2, 10).

Due to the linear pattern of the burns post MRI, the most likely causes of these burns were patient positioning and/or ECG lead placement. Burns associated with ECG electrodes, and pulse oximetry are more commonly seen in a circular pattern (17). The linear pattern of burns identified in our case cohort has been noted in people with MRI burns from ECG leads that are worn or have insufficient insulation from skin contact with blankets or sheets (18–20). Linear appearing skin to skin burns have also been reported along the line of contact between the thighs (7, 15) and presumptively this could have occurred in our cohort along the cubital fossa if the forelimbs were bent. It should be noted that 3 of the 4 dogs with cubital fossa burns were thin coated breeds (Doberman, Great Dane) which may have predisposed them to burns in this area, however, one of the most severe burns we discovered was on a Bernese Mountain Dog which has a long fur coat. Additionally, Doberman’s and Great Danes are more predisposed to cervical spondylomyelopathy and were more likely to have cervical vertebral column MRI (21). This phenomenon of skin to skin contact on the same appendage potentially causing MRI burns has previously been suggested in a case report in the veterinary literature (15).

The four dogs with the cubital and forelimb burns were all placed in the split head coil in dorsal recumbency with possible bending of the forelimbs causing a closed loop or contact with the coil. The single dog imaged on the CTL coil had a wound along the caudal thigh musculature. This was most likely due to the placement of the ECG lines within the coil with skin contact or trapping the ECG line between the patient and the coil. Based on the proposed mechanisms of MRI burns, the most likely cause of the burns in these patients was resonance circuit heating or the antenna effect. It has been demonstrated *in vitro* that significant enough heat can be generated by both mechanisms to cause the severe burns seen in these dogs (2).

Increased risk of MRI burns in people include anesthesia/sedation of the patient, longer duration of the MRI, and higher body mass index (BMI) (10, 19). It is interesting to note that 4 out of the 5 dogs were large breed, and similar to people, they may have increased risk due to larger body habitus despite having appropriate body condition scores. It should also be noted that no cats met inclusion criteria which also may be related to their smaller size. The use of general anesthesia prevents any indication of ongoing pain response and increases the risk of having an undetected area of heat. Therefore, in veterinary patients, the standard of care using anesthesia for MRI does increase the possibility of undetected injury in the acute phase similar to what was identified in this study. Never the less, patients that are awake can develop severe full thickness burn injuries without an immediate nociceptive response during the MRI due to the pattern and location of heating. This is believed to be due to the initial damage occurring deep to the skin and subsequently moving outward and the lack of sensory nerve sensation below the dermis. Human patients with MRI burns will often not exhibit clinical signs of pain for over 24 h and can have delayed appearance of visual signs of the burn (20, 22). Similarly we also noted a delay in time to recognition of burns in our cohort with a delay identified between soft tissue swelling in isolation and full declaration of burns, some of them full thickness. In addition, in the most severely affected patients the full extent of tissue damage took several days to fully declare due to the extensive depth of the burns.

The time for the MRIs in these dogs ranged from 19 to 45 min (average 32 min) and all had standard MRI protocols (T1 and T2 parasagittal and transverse imaging sequences) with no addition of lesser utilized or specialized sequences. Therefore, it was less likely these factors contributed to the patients burns. The majority of the noted injuries and the most severe burns were noted to be performed afterhours. The absence of more experienced diagnostic imaging technical staff may have resulted in less experienced individuals placing the ECG leads with decreased protective precautions or positioning patients in a less safe manner.

Based on the occurrence of these suspected MRI burns, the MRI positioning and setup protocols were reviewed at our institution and additional after-hours training was subsequently performed. The positioning and setup protocol was modified with an emphasis on coil selection, monitoring equipment setup, patient positioning, and the use of foam padding. This was done in an effort to ensure that there was no further potential for skin-skin contact such as limbs being bend and that monitoring equipment had at least 1–2″ of foam insulation between any leads and the skin (20). MRI safety recommendations in people have been documented in numerous publications (9, 17, 20). Recommendations include screening patients for metallic implants, positioning patients to avoid skin to skin contact, avoiding coiling of cables, avoiding skin contract with cables, and insulating the patient with manufacturer provided padding (9, 17, 20). In the years following this instructional change, no further burns have been noted, but the risk of MRI burns is still a potential complication of this diagnostic that clinicians need to be aware of.

**Figure 1:**
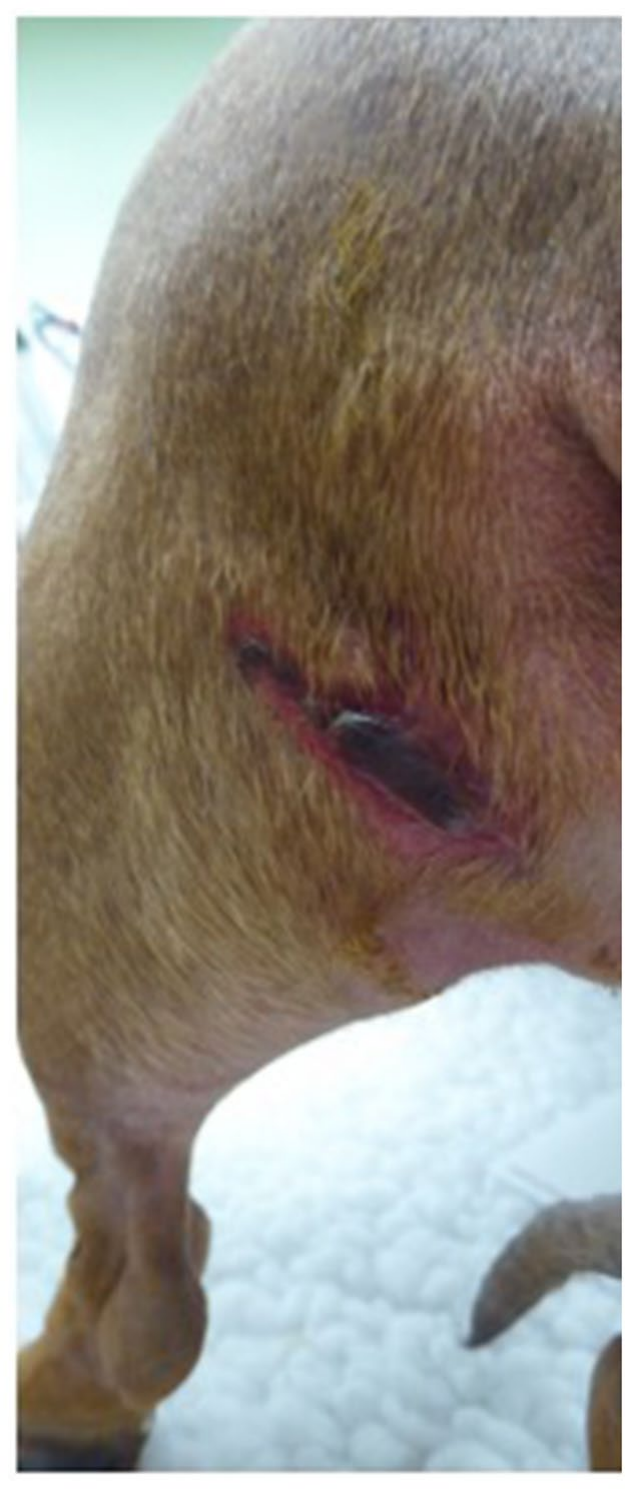
Full thickness burn on left caudal thigh area.

**Figure 2:**
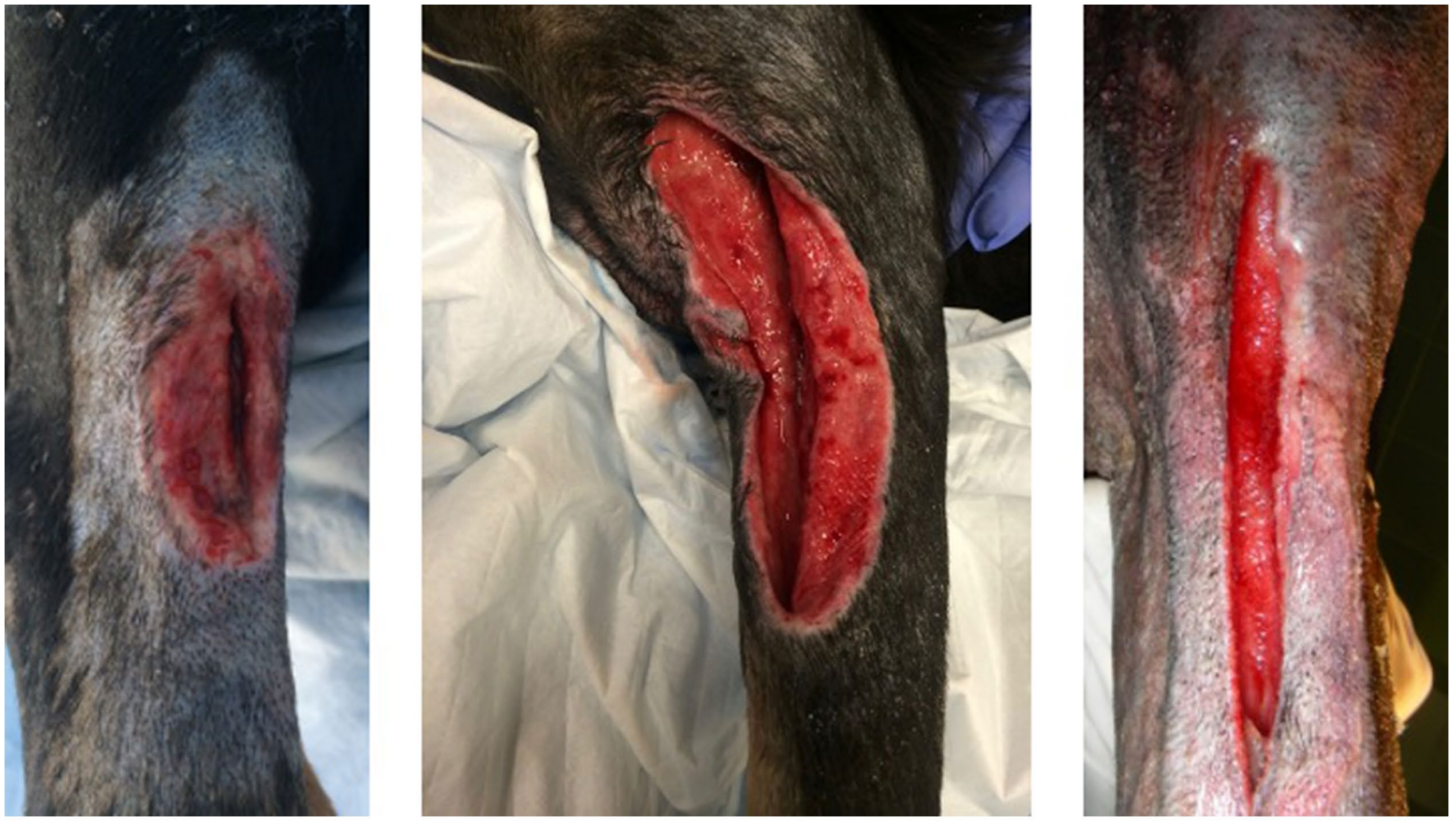
Severe full thickness burns in cubital region of 3 dogs.

The appearance of the wounds in these patients were all within a time frame noted in humans post MRI burn development (10, 20, 22, 23). The burns also had a very consistent linear pattern despite varying ranges of depth (Figure 1). It is also worth noting that the initial appearance in most cases was subcutaneous swelling and erythema that in the more severe insults progressed to extensive eschars over several days. In the three dogs with extensive full thickness burns, there was a distinct linear demarcation of loss of all tissue extending down to the bone (Figure 2). The wounds were determined to be MRI burns due to exclusion of other potential causes such as a thermal contact burns from a heating pad or severe thrombophlebitis. None of the patients had exposure to any other heating devices that would cause a linear burn in those locations, and none had consistent IV access in the distal vessels in those locations. In all cases, the patients were initially treated with standard of care topical wound treatments. Two dogs with the less severe burns were left to heal via second intention while the other three dogs with severe full thickness wounds were healed via tertiary intention to allow full declaration of the tissue damage and development of a healthy granulation bed prior to delayed primary closure. In these three dogs, there was substantial scarring and loss of mobility in the elbow joints. This loss of mobility and the morbidity associated with the burns in these dogs undoubtedly effected their neurologic rehabilitation and may have led to delayed return to function.

During the time period reviewed our institution performed 14,000 MRI exams making our MRI burn complication rate 0.035%. While there were a low number of cases consistent with MRI burns found in this study, some cases may have been missed not only due to the nature of retrospective studies, but also due to the potential lack of recognition by clinicians. Patients not detected in this study may have also experienced mild–moderate superficial burns that were attributed to other causes or hidden by the patients’ fur. Despite the full recovery of the burn wounds in these dogs, the severity of the injuries created significant clinical setbacks due to delays in their neurological rehabilitation and financial hardships due to prolonged and intensive wound care. Therefore, it is essential to recognize the potential, and the clinical presentation of MRI burns in veterinary medicine, and to institute and consistently review MRI safety protocols. That being stated the full mechanisms and physics of MRI burns is not fully understood to the degree to completely prevent their occurrence (14).

## Data Availability

The raw data supporting the conclusions of this article will be made available by the authors, without undue reservation.
